# Effect of Inoculation Treatment on Number of Primary Austenite Grains in Hypoeutectic Chromium Cast Iron: EBSD Imaging and Mathematical Structure Prediction

**DOI:** 10.3390/ma15186318

**Published:** 2022-09-12

**Authors:** Dorota Siekaniec, Dariusz Kopyciński, Edward Guzik, Andrzej Szczęsny

**Affiliations:** Faculty of Foundry Engineering, AGH University of Science and Technology, al. A. Mickiewicza 30, 30-059 Kraków, Poland

**Keywords:** primary austenite grains, chromium carbides, inoculation, EBSD imaging, mathematical structure prediction

## Abstract

This study proved the influence of an inoculation substance on the primary structure of chromium-cast iron. The inoculation procedure has developed very well in the field of grey cast iron production and mainly concerns the crystallisation of graphite eutectic grains in this material. However, in chromium cast iron, the inoculation problem is not well-recognised due to the formation of chromium carbides in white cast iron. One can easily increase the number of carbides in the cast iron’s structure, but this procedure will not always bring the expected benefits in terms of increasing the overall mechanical properties. In the research included in this publication, an experiment was carried out with the use of ferrotitanium as an inoculant for chromium-cast iron. As a result of using the EBSD (electron backscatter diffraction analysis) imaging method, it was proven that the Fe–Ti interaction significantly influenced the primary structure of chromium cast iron that was formed by austenite grains. The paper presents the growth laws of primary austenite grains in modified hypoeutectic chromium cast iron depending on the degree of supercooling, ΔT, and the amount of the Fe–Ti inoculant M. The results of the research made it possible to predict the structure of hypoeutectic chromium cast iron after Fe–Ti inoculation treatment. The article proves that the use of the inoculant can change the primary structure of chromium cast iron, increasing its impact strength by more than three times.

## 1. Introduction

**Figure 1:**
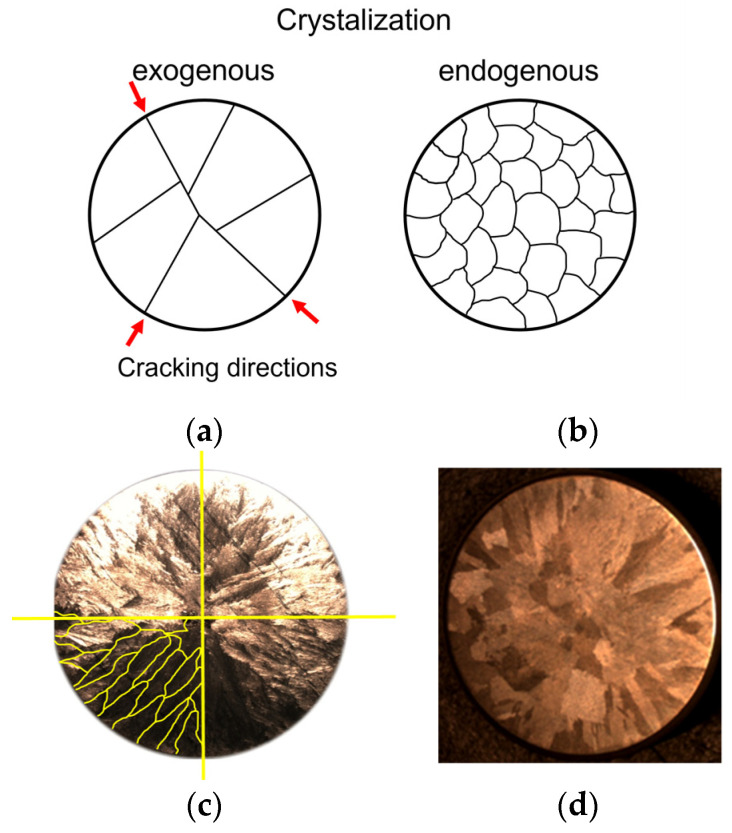
(**a**) Scheme [22,23] of coarse-grained microstructure formed during directional crystallisation with direction of cracking; (**b**) fine-grained microstructure formed during volumetric crystallisation; (**c**) surface of a metallographic specimen made of high-chromium cast iron with revealed directed macrostructure of grains of primary austenite with grain boundaries (marked with grain boundaries); (**d**) appearance of a sample made of the same cast iron but with equiaxed grains of primary austenite.

A very important issue that is related to the control of chromium cast iron’s structure is the introduction of additional elements to modify the structure. In the literature, one can find works that are related to the influence of titanium, vanadium, boron, bismuth [1,2,3,4,5,6,7,8,9,10,11,12], niobium [9,13], tungsten [14,15,16], and molybdenum [17,18] on the structure and properties of chromium cast iron. However, a distinction must be made between the introduction of elements into the alloy to modify the microstructure and the inoculation procedure to increase grain refinement and, thus, improve the mechanical properties of the product. Moreover, one question should be asked: is the inoculation process in white cast iron as important as it is in the case of grey cast iron? [19]. It should be remembered that the inoculation procedure in grey cast iron gives very good results as related to the improvement of the mechanical properties of the obtained products. The authors of this study have previously published papers [20,21,22] that have indicated that a well-chosen inoculant for chromium cast iron should increase the mechanical properties of this material. However, the most important achievement seems to be the elimination of defects that often occur in this material, i.e., porosity and hot cracks. In addition, it should also be mentioned that white cast iron (including chromium cast iron) has a tendency to directionally crystallise from the mould wall; in such a case, this will lead to the appearance of porosity in various places of the casting. The inoculation process can help avoid this type of defect. A very good scheme of the mechanism that we can follow when choosing an inoculant is shown in [23] and in Figure 1; this was developed on the basis of [24] and concerns the formation of primary austenite in white cast iron based on the so-called series of the types of the dendritic crystallisation of primary austenite grains in white cast iron. Figure 1a shows that the coarse-grained structure may lead to fractures of the casting along the boundaries; theoretically, grain fragmentation (Figure 1b) should counteract such a phenomenon. Figure 1c shows the actual microstructure that was revealed on the surfaces of metallographic specimens of chromium cast iron in which the grains crystallised directionally. Figure 1d shows this crystallisation being formed equiaxially.

**Figure 2:**
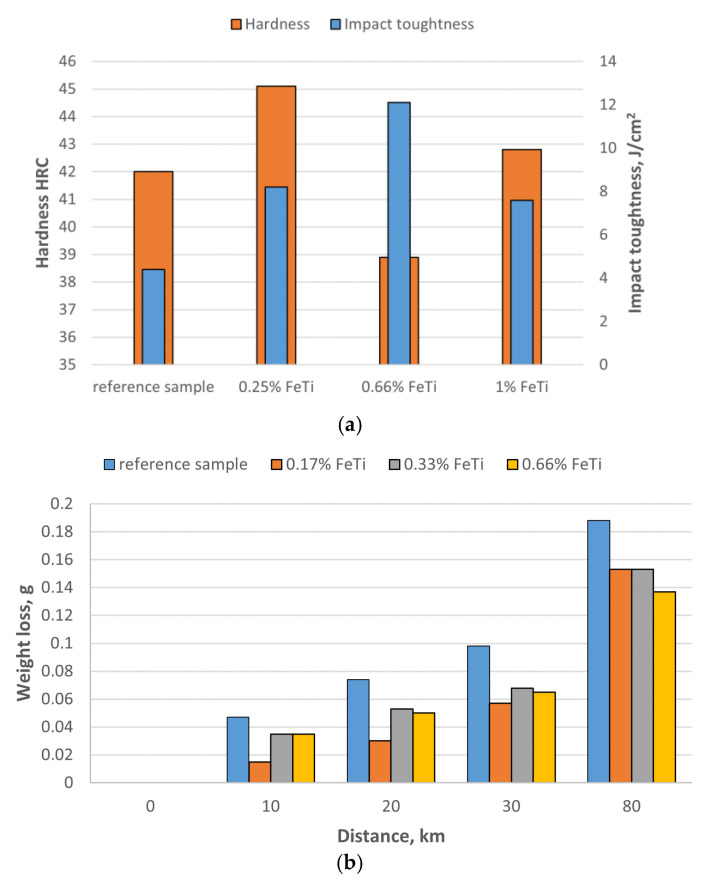
(**a**) Impact toughness and hardness of cast iron inoculated with Fe–Ti [22]; (**b**) results of wear resistance as function of distance [20].

In previous works [20,21,22,23], the authors proved that the addition of Fe–Ti to a molten alloy causes favourable changes in the mechanical properties of high-chromium cast iron. Inoculating chromium cast iron with fine Fe–Ti increases the impact toughness of this material and increases the wear resistance for a constant level of hardness (as shown in Figure 2).

**Figure 3:**
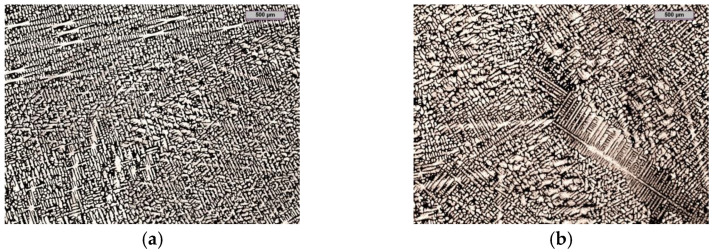

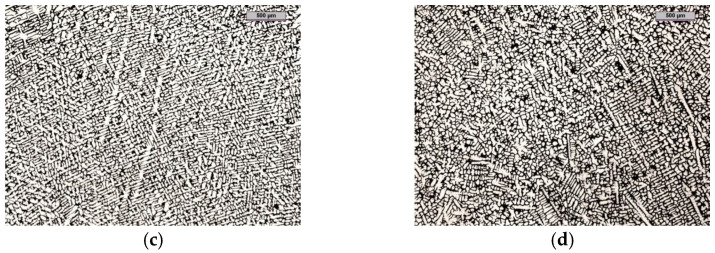
Chromium cast iron microstructure inoculated with (wt.%): (**a**) 0.0 Fe–Ti; (**b**) 0.17 Fe–Ti; (**c**) 0.33 Fe–Ti; (**d**) 0.66 Fe–Ti (magnification–25x).

In [20], the authors proved that the addition of an inoculant in the form of Fe–Ti significantly changed the degree of supercooling ΔT during the crystallisation of primary austenite, while it had a slight influence on parameter ΔT for the crystallisation of the carbide eutectic. This means that the inoculation with the use of Fe–Ti has a large effect on the increase of the number of primary grains and, at the same time, a small effect on the number of eutectic precipitates of chromium carbide. It can be concluded that, with increasing amounts of the inoculant, the number of dendrites that increase in a directional way decreases (Figure 3).

Increasing the amount of primary grains by applying the inoculation treatment can increase the impact toughness of chromium cast iron. As shown in Figure 2, the impact strength before inoculations is about 4 J/cm^2^ and after it increases almost threefold. Another way to increase the impact toughness of chromium cast iron is to add alloying additives. The cast iron described in [25], with the composition of 2.3 ÷ 2.9% C, 18 ÷ 22% Cr, 1.4 ÷ 2.0% Mo, 0.5 ÷ 0.8% Cu, 0.5 ÷ 0.8% Ni, had an impact strength of 10.3 J/cm^2^. However, the use of alloying additives significantly increases the production costs of castings. The correlation of the results presented in Figure 2 with the microstructures in Figure 3 is problematic due to the difficulty in determining the amount of primary grains using light microscopy. For this purpose, it was decided to use the EBSD method for grain identification and counting [26,27].

## 2. Thesis and Purpose of Work

In the research that is presented in this publication, the authors put forth the following thesis:

*Using the EBSD technique and obtaining an austenitic metal matrix in an inoculated hypoeutectic high-chromium cast iron, it is possible to:*

- *determine the growth law of primary austenite grains as a function of supercooling, N = f (ΔT);*
- *determine the amount of inoculant (Fe–Ti) as a function of the number of primary austenite grains, M = f (N).*

The aim of the research was to develop a mathematical description of the dependencies that allows us to predict the structure of hypoeutectic cast iron after applying an inoculation treatment with the Fe–Ti ferroalloy.

## 3. Methodology

Melts were carried out in the Foundry Components Research and Development Centre foundry located in Odlewnie Polskie S.A. in Starachowice, Poland. A medium-frequency induction furnace with a capacity of 120 kg was used for the tests. Molten metal was overheated to 1600 °C and transferred in a 30-kg-capacity ladle, and moulds were poured.

Casting moulds made of loose self-hardening sands (SMS) were used in the research. Plates with dimensions of 100 × 100 × 30 mm were cast for microstructure analysis. Castings of samples for impact testing with dimensions of 10 × 10 × 55 mm were also made. The chemical composition of the reference alloy as well as the chemical compositions of the inoculated cast iron samples and the values of the measured degree of supercooling ΔT for the crystallisation of primary austenite are presented in Table 1. Impact tests were performed using a Charpy Impact test machine., VEB WerkstoffprUfmaschinen, Leipzig, mass of the pendulum kg 6.764, work content of the pendulum kpm 5.

Samples were cut out, placed in mounting cups, and then poured over with acrylic resin. Then, using the Struers RotoPol-11 machine, they were ground using diamond discs 120, 220, 600, and 1200 (rinsed with water), and polished on the MD-Nap disc with the use of diamond suspension and lubricant. The samples were etched with Villela’s regent after rinsing in ethyl alcohol and drying.

From the cast plates, samples were cut out for analysing the primary microstructure; this was performed on an FEI Quanta 3D FEGSEM high-resolution scanning electron microscope with an additional FIB ion column and integrated with the EDAX Trident system (EDAX Genesis spectrometer, WDS Genesis LambdaSpec spectrometer, and retrograde electron acquisition system distributed by EBSD Genesis TSL) located at the Institute of Metallurgy and Materials Science of the Polish Academy of Sciences in Krakow, Poland.

The EBSD testing provides many results in graphical and numerical forms; one of the basic ones is an orientation map—a so-called Euler map. The easiest way to generate an orientation map is to draw the three Euler angles with an RGB legend. The colour Euler map gives basic information on the microstructure; however, it also has some limitations.

One of the most common occurrences is that small changes in orientation do not always correspond to changes in the colour of the scale (which can be confusing). To overcome this problem, a different orientation map is often used—an IPF (inverse pole figure) map. The IPF map uses a different colour scheme that is much easier to interpret and that usually does not have large colour changes for slight changes in orientation. The colour scheme is designed by specifying the colour of each corner of the so-called base triangle. For each map, a sample reference direction (e.g., rolling direction) will be selected, and a colour will be assigned based on the measured crystal orientation.

## 4. Microstructure Analysis Using EBSD Technique

The experience of the authors of the work shows that, when using the standard techniques of metallographic analysis, it is impossible to assess the primary structure of alloy cast iron castings. However, it should be emphasised that this structure has a great influence on the functional properties of these alloys. In order to reveal the primary austenite grains, the aforementioned EBSD technique was used. The same set of high-chromium cast iron samples of a hypoeutectic composition that were cut from a plate that was 30 mm thick were analysed. The analysis was carried out analogously to the one that is described in [28], so the IPF map of the crystallographic orientation of the unique grain colour map, the grain disorientation angles, and the map of the distribution of the occurring phases were determined.

### 4.1. Reference Cast Iron Sample

**Figure 4:**
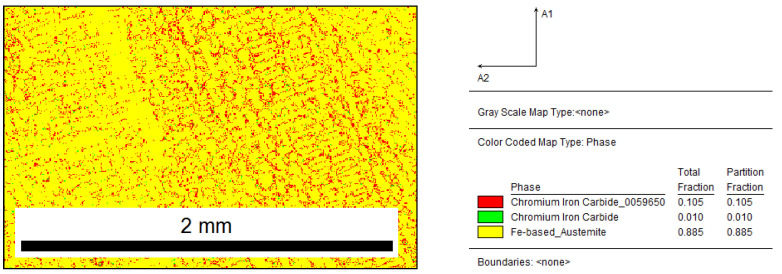
Reference sample: distribution map of occurring phases, red area—M_7_C_3_, green area—M_3_C.

The phase map presented in Figure 4 confirms the assumption that, for a given chemical composition of cast iron, the structure is mostly austenite (89%) and chromium carbides (11%).

**Figure 5:**
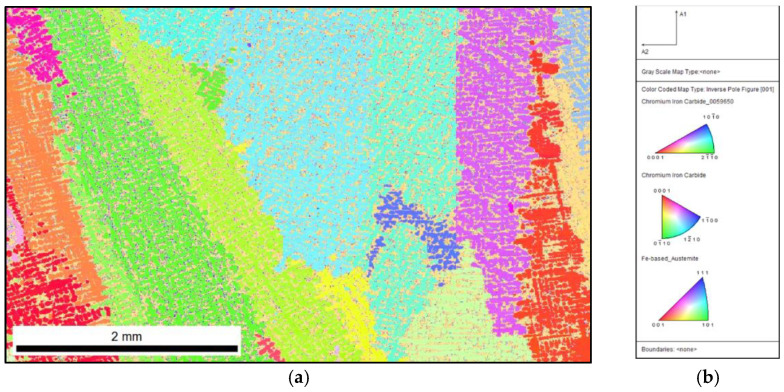
Reference sample: (**a**) IPF map with marked primary grains; (**b**) reference triangle.

From the point of view of the conducted research, the most important is the IPF map of the primary austenite grains that is presented in Figure 5. From this, the number of grains can be determined and then correlated with the degree of supercooling ΔT and the amount of the M inoculant that was applied.

### 4.2. Inoculated Cast Iron (0.17 wt.% Fe-Ti)

**Figure 6:**
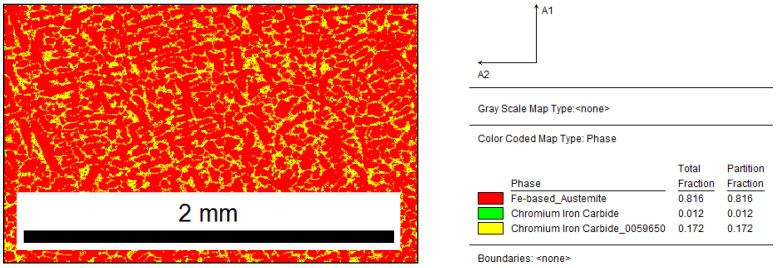
Inoculated sample with 0.17% Fe–Ti: distribution map of occurring phases, yellow area—M_7_C_3_, green area—M_3_C.

The phase-distribution map shown in Figure 6 shows that the sample structure contained austenite (82%) and chromium carbides (18%). Compared to the phase fraction for the sample before and after the modification, it can be observed that the introduction of Fe–Ti increased the number of carbides by 7%.

**Figure 7:**
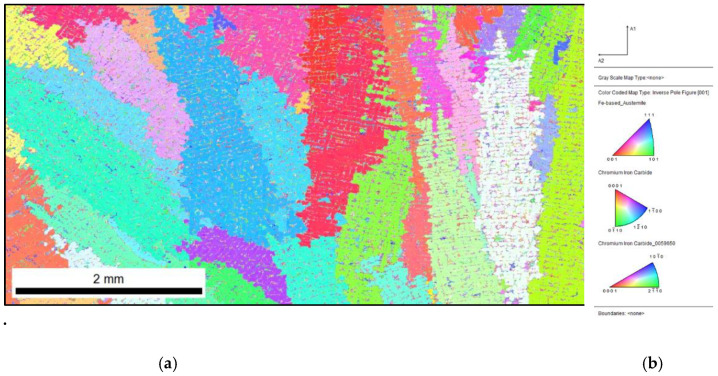
Inoculated sample with 0.17% Fe–Ti: (**a**) IPF map with marked primary grains; (**b**) reference triangle.

When comparing the unique colour map with the reference sample and the inoculated 0.17% Fe–Ti sample, no clear effect of the inoculation procedure can be observed on the microstructure, while the difference is visible in the analysis of the primary microstructure. By comparing the IPF map of the reference sample (Figure 5) with the inoculated sample (Figure 7), the fragmentation of the primary austenite grains can be observed. Inoculation with as little as 0.17% Fe–Ti contributed to the fragmentation of these grains.

### 4.3. Inoculated Cast Iron (0.33 wt.% Fe–Ti)

**Figure 8:**
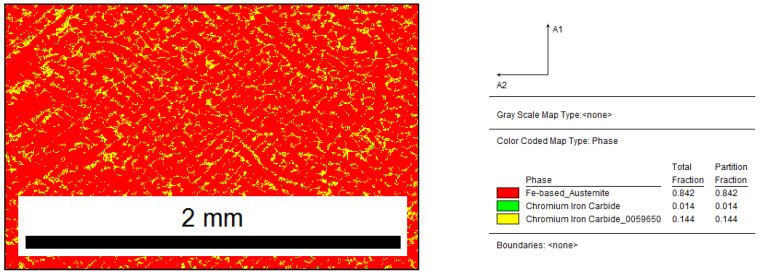
Inoculated sample with 0.33% Fe–Ti: distribution map of occurring phases, yellow area—M_7_C_3_, green area—M_3_C.

**Figure 9:**
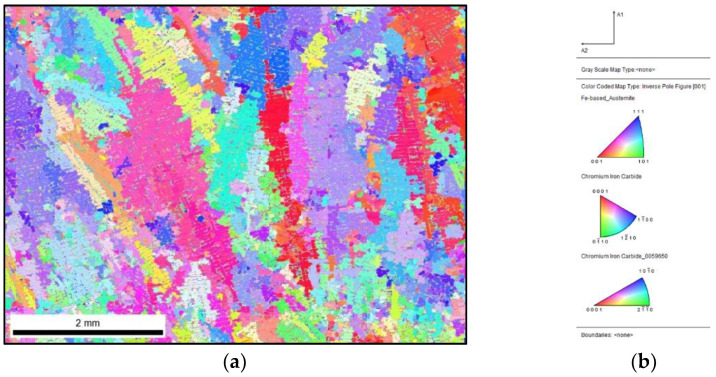
Inoculated sample with 0.33% Fe–Ti: (**a**) IPF map with marked primary grains; (**b**) reference triangle.

The analysis by the EBSD method for the sample that was inoculated with 0.33% Fe–Ti (presented in Figure 8 and Figure 9) showed that the inoculation affected the fragmentation of the microstructure. The structure of the casting consisted of 84% austenite and 16% chromium carbides. It should be noted that the use of the EBSD technique was not intended to detect titanium carbides in the microstructure but to merely check the thesis of increasing the number of primary austenite grains during the Fe–Ti inoculation.

When analysing the IPF map with marked primary grains (Figure 9), it can be seen that, as related to the previously analysed samples, the microstructure was finer. The priority directions of growth for the austenite grains were 111 and 001.

### 4.4. Inoculated Cast Iron (0.66 wt.% Fe-Ti)

**Figure 10:**
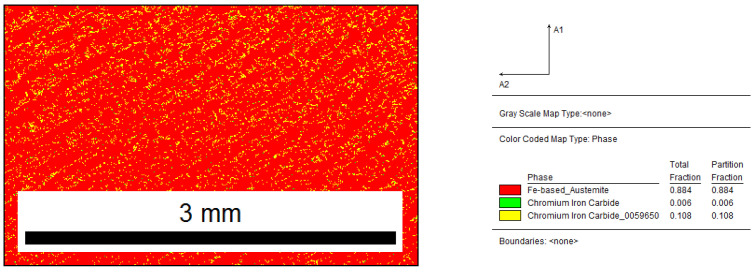
Inoculated sample with 0.66% Fe–Ti: distribution map of occurring phases.

In the phase composition that is shown in Figure 10, the austenite amounted to 88%, and the chromium carbides amounted to 12%.

**Figure 11:**
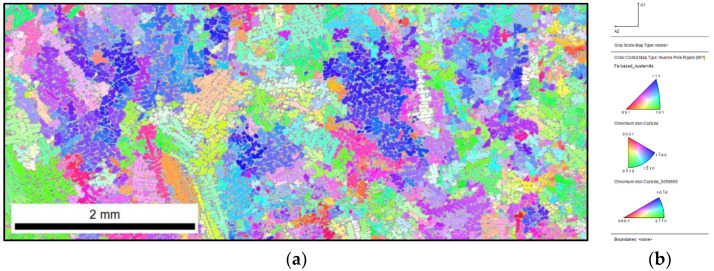
(**a**) Inoculated sample with 0.66% Fe–Ti: IPF map with marked primary grains; (**b**) reference triangle.

When analysing the charts above, it can be noticed that the inoculation of 0.66% Fe–Ti also influenced the fragmentation of the primary structure of the casting. From the IPF map that is presented in Figure 11, it can be seen that the addition of 0.66% Fe–Ti had the greatest effect on the fragmentation of the microstructure. Still, the most favoured direction of the austenite grain growth was toward the 111 direction; in this case, however, many more grains also grew toward 101 (which was rare in the earlier samples).

## 5. Determination of Number of Primary Austenite Grains

Due to the subject of the research, the most important results from the EBSD analysis were those that concerned the number of primary austenite grains. Correlating this data with the amounts of the inoculant that were employed will be used to determine the primary austenite grain growth law for the inoculated hypoeutectic high-chromium cast iron. Based on the IPF maps, the number of grains for each sample was determined. Figure 5 represents the reference sample. The drawings in Figure 7, Figure 9, and Figure 11 show the microstructures of the reference samples that were inoculated with 0.17, 0.33, and 0.66% Fe–Ti, respectively. As can be seen from the analysis of the microstructures, the Fe–Ti inoculation fragmented not only the chromium carbides but also the primary austenite grains. Along with increasing the mass of the ferrititanium as an inoculant to be introduced into the molten metal, the final result was a casting structure that was characterised by greater numbers of primary austenite grains and chromium carbides. For each sample, the number of N grains in a given area of metallographic sample P was calculated according to Formula (1) while taking the N_W_, N_P_, and N_R_ grains into account; then, the surface density of the N_A_ grains (2) and the N_V_ volumetric grain density (3) were calculated according to the formula.

The results of the calculations are presented in Table 2. Stereological Equation (3) can be used to calculate spatial grain number Nv, which should give the average number of grains of primary austenite per unit volume. During the calculations, the following assumptions were made so that the spatial grain configurations followed the so-called Poisson–Voronoi model [29], as shown in Equations (1)–(3).

(1) $$N=N_{W}+0.5N_{P}+0.25N_{R}$$

(2) $$N_{A}=\frac{N}{P},\;\frac{1}{{\mathrm{mm}}^{2}}$$

(3) $$N_{V}=0.568\;\Delta {\left(N_{A}\right)}^{\frac{3}{2}}\;,\frac{1}{{\mathrm{mm}}^{3}}$$

where:

N—number of grains in area
N_W_—number of whole grains in area
N_P_—number of grains cut by sides of area
N_R_—number of grains in corners of area
N_A_—surface grain density, $\frac{1}{{\mathrm{mm}}^{2}}$
P—area, mm^2^
N_V_—volumetric grain density,$\;\frac{1}{{\mathrm{mm}}^{3}}$

**Table 2:** Data and results for calculating numbers of N_A_ and N_V_ grains.

Sample	N	P	N_A_	N_A_	N_V_	N_V_
		mm^2^	1/mm^2^	1/cm^2^	1/cm^3^	1/m^3^
Reference	6.5	17.1403	0.3792	37.92	132.64	1.33 × 10^8^
Inoc. 0.17% Fe–Ti	22.75	20.1416	1.1295	112.95	681.84	6.82 × 10^8^
Inoc. 0.33% Fe–Ti	174.5	27.4176	6.3645	636.45	9120.05	9.12 × 10^9^
Inoc. 0.66% Fe–Ti	233	16.2031	14.3799	1437.99	30,973.04	3.10 × 10^10^

The calculations concern the mathematical description of the effect of an Fe–Ti inoculant addition on the structure of cast iron. An analysis of the experimental data shows that the increase in the inoculant’s share significantly influenced the final refinement of the casting’s microstructure (Table 2). At the same time, it can be observed that the value of supercooling ΔT decreases when the share of inoculant M increases (Table 1). This is due to the fact that the addition of the inoculant increases the number of heterogeneous nucleation sites. As a result, an increased number of austenite grains appear—even at lower sub-cooling values. The discussed process affects the heat balance in the solidifying alloy: the appearing grains are accompanied by an exothermic thermal effect (which is then intensified by their further growth). This has an effect on the recalescence that can be observed in the cooling curve (the maximum sub-cooling as read from the course of this curve). This process coincides with the conclusions that can be led by an analysis of the theoretical formula for the critical dimension of the nucleus—Equation (4). The foundations of the nucleation theory were developed by Volmer and Weber [30] and then further developed by Turnbull [31]. The transformation of Equation (4) allows us to determine the degree of supercooling ΔT that is required to reach the critical radius. This radius is closely related to the size of the rootstock on which the germination process takes place. If the share of nucleation sites with larger radii increases, then more stable grains will appear for lower supercooling ΔT—the growth of which generates the heat of the crystallisation. The more the grains grow, the more heat is released due to the crystallisation [30,31], as shown in Equation (4).

(4) $$r^{*}=\frac{2\;\sigma_{l,s}}{\Delta S\;\Delta T}$$

where:

- σ_1,s_—surface tension at liquid–solid interface, $\frac{J}{m^{2}}$
- ΔS—entropy change, $\frac{J}{m^{3}K}$
- ΔT—degree of sub-cooling, K

**Figure 12:**
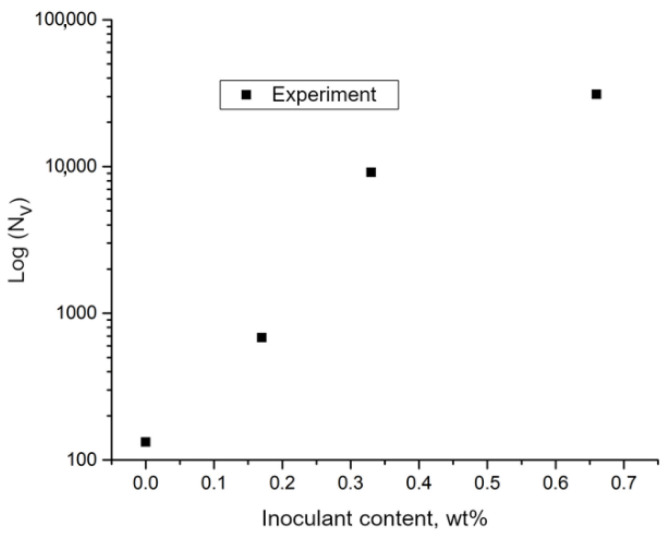
Results of grain density measurement presented on logarithmic scale.

The Fe–Ti-inoculant addition on the austenite grain density, the approximation using a two-parameter exponential function was used. Equations (5)–(8), presented below, are the result of elaborating the obtained data in which the relationships related to Nv and ΔT were determined. The choice of such a function was dictated by the nature of the distribution of the experimental points in the coordinate system in which a logarithmic scale was used on the ordinate axis (Figure 12).

Despite the slight deviations, it can be observed that the results of the experimental measurements are arranged in a straight line after using the logarithmic scale. In connection with the presented observation, a curve approximation was used in the following form (Equation (5)):

(5) $$N_{V}\left(M\right)=Aexp\left(BM\right),\;m{}^{-3}$$

where:

M—amount of inoculant, wt.%
A and B—matching parameters

The following values of the fit parameters were determined: A = 1462 × 10^6^, and B = 4.635. The formula of the function that describes the effect of the modifier addition on the volumetric density of the austenite grains is as follows (Equation (6)):

(6) $$N_{V}\left(M\right)=1462\cdot 10^{6}\exp(4.635M),\;m{}^{-3}$$

**Figure 13:**
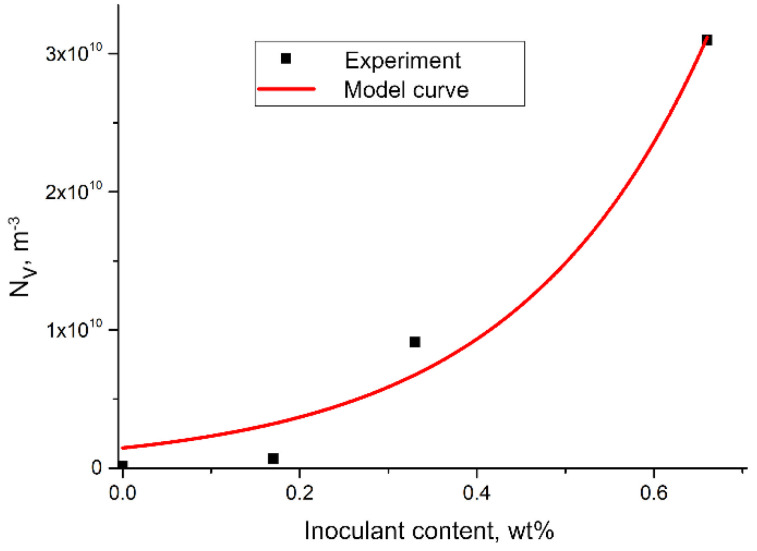
Effect of addition of Fe-Ti inoculant on austenite grain density — the results of experiment and model curve calculated from (3).

The matching factor was R^2^ = 0.97. A graph of the model curve is presented in Figure 13.

In order to determine the relationship between the maximum degree of the supercooling and the grain density, the assumption about the exponential nature of this relationship was again made. The approximation was therefore aimed at determining fit parameters C and D in the following Equation (7):

(7) $$N_{V}\left(\Delta T\right)=Cexp\left(D\Delta T\right),\;m{}^{-3}$$

where:

1. ΔT—maximum degree of sub-cooling, K;
2. Parameters C and D—adjustment parameters.
3. The following values of the adjustment parameters were determined: C = 1.44 × 10^11^, and D = −0.31.

As a result of the calculations, the following Equation (8) was obtained:

(8) $$N_{V}\left(\Delta T\right)=1.44\cdot 10^{11}\exp(-0.31\Delta T),\;m{}^{-3}$$

**Figure 14:**
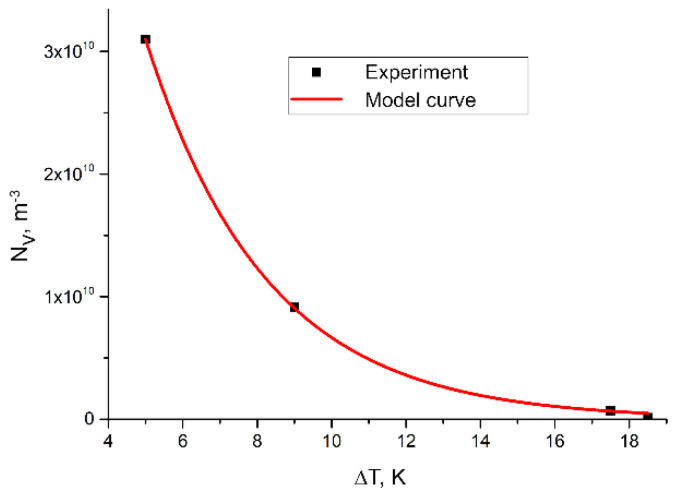
Influence of maximum degree of supercooling ΔT on austenite grain density—the results of experiment and model curve calculated from (5).

This dependence is shown in Figure 14.

Impact resistance tests of the obtained cast iron were carried out. The results are presented in Table 3.

By comparing the results presented in Table 2 and Table 3, it can be concluded that there is an optimal amount of the inoculant which, when introduced into the chromium cast iron, will significantly increase the impact toughness. This is due to the formation of an appropriate number of austenite primary grains during the crystallisation of molten metal.

## 6. Conclusions

The conducted research and analysis allowed for the following conclusions:

- Inoculation of chromium cast iron using Fe–Ti increases the number of carbides by 7%;
- Inoculation of chromium cast iron using Fe–Ti significantly increases the number of primary austenite grains;
- Research allowed us to determine an exponential function describing the effect of the addition of Fe–Ti (M) inoculant on the volume density of primary austenite grains in the following form: $N_{V}\left(M\right)=1462\cdot 10^{6}\exp(4.635M)$, m^−3^;
- Research allowed us to determine a relationship between the maximum degree of supercooling ΔT and the density of primary austenite grains of exponential nature in inoculated high-chromium cast iron: $N_{V}\left(\Delta T\right)=1.44\cdot 10^{11}\exp(-0.31\Delta T)$, m^−3^;
- For chrome cast iron without inoculation, the impact strength does not exceed 4 J/cm^2^;
- With the optimal content of the inoculant of 0.33% Fe–Ti, the impact strength of cast iron is 14.4 J/cm^2^

## Figures and Tables

**Table 1:** Chemical composition of chromium cast iron and values of degrees of supercooling ΔT for crystallisation of primary austenite.

Inoculant Addition	C	Si	Mn	P wt.%	S	Cr	Ti	ΔT K
+0.0% Fe–Ti	1.81	0.77	0.47	0.02	0.01	21.19	0.0036	18.5
+0.17% Fe–Ti	1.78	0.79	0.47	0.02	0.01	21.21	0.0599	17.5
+0.33% Fe–Ti	1.77	0.77	0.42	0.02	0.01	21.21	0.0882	9.0
+0.66% Fe–Ti	1.67	0.77	0.43	0.02	0.01	21.06	0.2043	6.0

**Table 3:** Impact strength of the tested cast iron samples.

Inoculant Addition	Impact Strength J/cm^2^
+0.0% Fe–Ti	4.4
+0.17% Fe–Ti	8.0
+0.33% Fe–Ti	14.0
+0.66% Fe–Ti	12.1

## Data Availability

Not applicable.
